# Supplementary material for: Phytochemical Profile, Antioxidant Capacity, and Photoprotective Potential of Brazilian Humulus Lupulus
Source: Pharmaceuticals (Basel). 2025 Aug 20;18(8):1229. doi: 10.3390/ph18081229 (PMC12389064; doi:10.3390/ph18081229)
Supplement: Supplementary file 1 [file pharmaceuticals-18-01229-s001.zip › pharmaceuticals-3756833-supplementary.docx]

**Supplemental File**

**Table S1.** Analytical curve equation and coefficient of determination (R^2^) of compounds detected in *Humulus lupulus* L. leaves crude extracts from Cascade, Columbus, Comet and Nugget varieties.

| **Compound** | **Analytical curve equation** | **R^2^** |  | |
| --- | --- | --- | --- | --- |
| Clorogenic acid | y = 2265,823x - 21644,460 | 0.996 |  |  |
| Sinapic acid | y = 969,0258x + 9042,887 | 0.986 |  |  |
| Protocatechuic acid | y = 1428,893x - 5939,097 | 0.994 |  |  |
| *p*-Hydroxybenzoic acid | y = 1903,034x + 1133,0310 | 0.998 |  |  |
| *p*-Coumaric acid | y = 3426,103x - 14433,060 | 0.997 |  |  |
| Caffeic acid | y = 3786,001x - 24911,060 | 0.998 |  |  |
| Ferulic acid | y = 737,7287x - 7004,621 | 0.992 |  |  |
| Quinic acid | y = 1818,244x - 5402,542 | 0.990 |  | |
| Malic acid | y = 1793,454x - 16836,25 | 0.992 |  | |
| Nicotinic acid | y = 208,0211x - 1554,629 | 0.995 |  | |
| Catechin | y = 2644,606x - 25730,99 | 0.995 |  | |
| Epicatechin | y = 1146,058x - 12562,15 | 0.990 |  | |
| Quercetin | y = 5080,717x + 119391,4 | 0.991 |  | |
| Rutin | y = 4219,125x - 8297,430 | 0.999 |  | |
| Luteolin | y = 1880,476x + 69297,92 | 0.988 |  | |
| Naringenin | y = 6281,776x + 59537,760 | 0.993 |  | |
| Hydroxybenzaldehyde | y = 2467,528x - 12200,660 | 0.989 |  | |
| Vanillin | y = 344,7863x + 74949,510 | 0.990 |  | |
| Isovaniline | y = 9407,413x + 957820,800 | 0.996 |  | |
| Syringaldehyde | y = 2340,794x - 775,453 | 0.992 |  | |
| Caffeine | y = 2951,902x - 12594,490 | 0.990 |  | |

**Chromatograms**


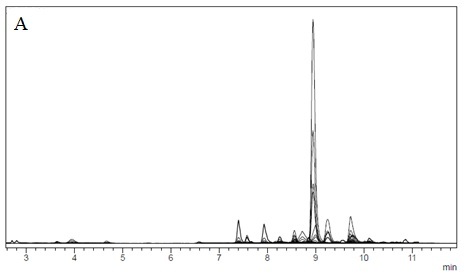


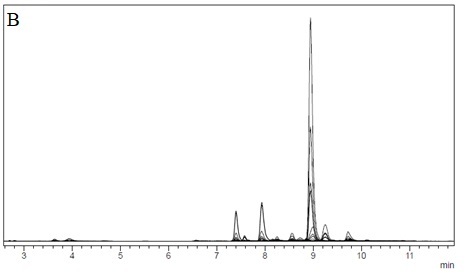


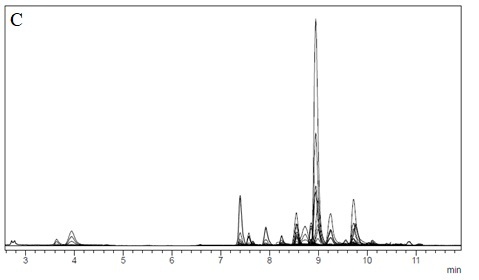


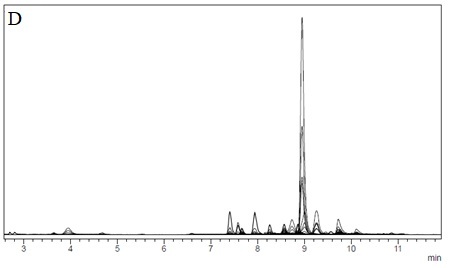


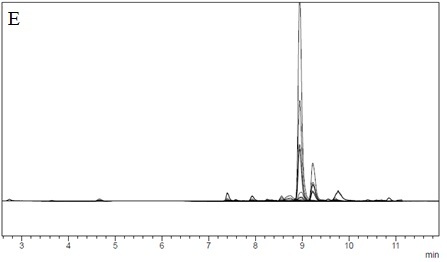


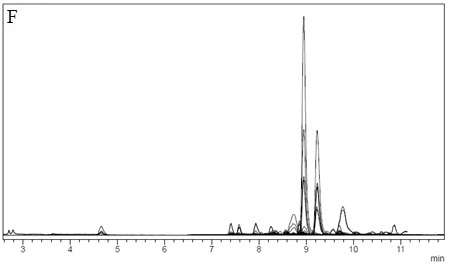


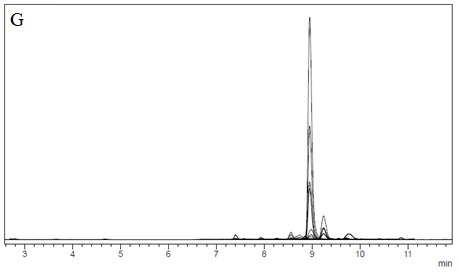


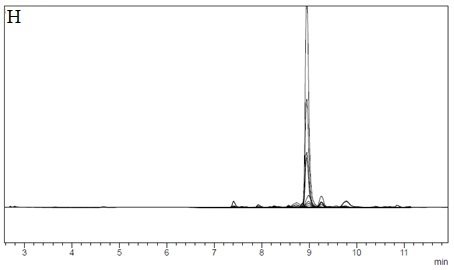


Figure S1 – Chromatograms of compounds detected by UHPLC-MS/MS of the crude extracts of *H. lupulus* cones and leaves. A) Cone cascade; B) Cone Columbus; C) Cone Comet; D) Cone Nugget; E) Leaves Cascade; F) Leaves Columbus; G) Leaves Comet; H) Leaves Nugget.
